# Lung-Derived Mediators Induce Cytokine Production in Downstream Organs via an *NF*-**κ** *B*-Dependent Mechanism

**DOI:** 10.1155/2013/586895

**Published:** 2013-03-27

**Authors:** E. K. Patterson, L. J. Yao, N. Ramic, J. F. Lewis, G. Cepinskas, L. McCaig, R. A. W. Veldhuizen, C. M. Yamashita

**Affiliations:** ^1^Lawson Health Research Institute, London, ON, Canada N6A 4V2; ^2^Department of Medicine, Physiology and Pharmacology, Western University, London, ON, Canada

## Abstract

In the setting of acute lung injury, levels of circulating inflammatory mediators have been correlated with adverse outcomes. Previous studies have demonstrated that injured, mechanically ventilated lungs represent the origin of the host inflammatory response; however, mechanisms which perpetuate systemic inflammation remain uncharacterized. We hypothesized that lung-derived mediators generated by mechanical ventilation (MV) are amplified by peripheral organs in a “feed forward” mechanism of systemic inflammation. Herein, lung-derived mediators were collected from 129X1/SVJ mice after 2 hours of MV while connected to the isolated perfused mouse lung model setup. Exposure of liver endothelial cells to lung-derived mediators resulted in a significant increase in G-CSF, IL-6, CXCL-1, CXCL-2, and MCP-1 production compared to noncirculated control perfusate media (*P* < 0.05). Furthermore, inhibition of the NF-**κ**B pathway significantly mitigated this response. Changes in gene transcription were confirmed using qPCR for IL-6, CXCL-1, and CXCL-2. Additionally, liver tissue obtained from mice subjected to 2 hours of *in vivo* MV demonstrated significant increases in hepatic gene transcription of IL-6, CXCL-1, and CXCL-2 compared to nonventilated controls. Collectively, this data demonstrates that lung-derived mediators, generated in the setting of MV, are amplified by downstream organs in a feed forward mechanism of systemic inflammation.

## 1. Introduction

Acute lung injury (ALI) and the acute respiratory distress syndrome (ARDS) represent a spectrum of diseases characterized by the rapid onset of pulmonary infiltrates and progressive hypoxemia in the absence of significant left ventricular dysfunction [1]. Within the early phases of ALI, the role of mechanical ventilation and its influence on patient outcomes has been an area of specific interest [2]. It is now widely acknowledged that the use of excessive tidal volumes in patients with underlying ALI can further perpetuate lung dysfunction [3] while limiting injury through the use of lower tidal volumes has been the only therapeutic maneuver shown to improve survival [4]. Furthermore, the proinflammatory response associated with the mechanical stress of ventilation, known as biotrauma [5], represents one of the key mechanisms by which mechanical ventilation may be critical in determining patient outcomes. 

Prior studies have demonstrated that the injured lung serves as the primary origin of proinflammatory mediators which may decompartmentalize into the systemic circulation [6–8]. Recent studies from our laboratory have shown that these lung-derived mediators are capable of eliciting the expression of surface adhesion molecules in liver endothelial cells both directly and in a tidal volume-dependent fashion [9, 10]. From a clinical perspective, it has been demonstrated that patients ventilated with low-tidal volumes had a reduction in plasma proinflammatory mediator levels compared to those patients ventilated by conventional strategies and, notably these levels correlated with a reduction in multiple organ failure [11]. Although such evidence implicates the lung as the primary source of mediators leading to systemic inflammation, the specific mechanisms that serve to perpetuate and propagate the ensuing proinflammatory signaling cascade remain uncharacterized. 

For example, it remains unknown, whether the marked rise in plasma cytokines can be attributed entirely from a “spillover” phenomenon of a mechanically ventilated, injured lung to the systemic circulation or whether a primary inflammatory signal generated by the lung may be secondarily amplified by downstream peripheral organs. Therefore, characterization of the discrete signaling processes which drive persistent increases in systemic inflammatory mediators and the localization of their specific cellular origins may be critical in the development of effective therapeutic agents aimed at mitigating the inflammatory response resulting from mechanical ventilation. 

One of the intracellular signaling pathways most widely recognized for its importance in inflammation is the nuclear factor kappa B (NF-*κ*B) signaling pathway. It has been well established that many receptors activate the NF-*κ*B pathway, the most extensively studied of which are the interleukin (IL), tumor necrosis factor (TNF), and toll-like receptor families [12]. The “canonical” activation of the NF-*κ*B pathway involves phosphorylation of p65 (RelA), and its translocation to the nucleus [13] leading to a number of proinflammatory responses including the upregulation of adhesion molecules (on both endothelial cells and leukocytes) and transcriptional regulation of a wide array of cytokines and chemokines [12]. Although activation of the NF-*κ*B pathway may be involved in the resolution of inflammation, particularly through its “alternative” pathway, we describe studies involving the acute phase of inflammation wherein the proinflammatory actions of NF-*κ*B activation predominate [12].

In the current study, it was hypothesized that inflammatory mediators generated by the lung in response to mechanical ventilation are secondarily amplified by downstream organs in a “feed forward” mechanism of systemic inflammation. Herein, we demonstrate that lung-derived mediators are definitively upregulated by liver tissues in both *in vitro* and *in vivo* models of mechanical ventilation-induced inflammation. Further studies examining specific intracellular pathways responsible for mediator amplification demonstrate that activation of the inflammation relevant NF-*κ*B signaling pathway in liver endothelial cells is in part responsible for these observations.

## 2. Materials and Methods 

### 2.1. Study Design

In order to obtain inflammatory mediators generated and released specifically from the lung into the systemic circulation, the isolated perfused mouse lung (IPML) model was employed. Lungs were mechanically ventilated using the *ex vivo* IMPL setup and lung perfusate was obtained after a completion of the ventilation protocol. Subsequently, mouse liver endothelial cells were exposed to lung perfusate to determine whether subsequent increases in inflammatory mediators were observed and the signaling processes that may be involved such as the inflammation-associated NF-*κ*B pathway. Furthermore the physiological relevance of these *ex vivo *and *in vitro* studies was validated using an *in vivo* model of mechanical ventilation to observe similar findings in intact whole liver tissues.

### 2.2. Animals

Male mice were used for experiments (Charles River, Saint-Constant, Canada). All procedures were approved by the Animal Use Subcommittee at the University of Western Ontario in agreement with the guidelines of the Canadian Council of Animal Care. All animals were acclimatized a minimum of 72 hours prior to use in the experiments and had free access to water and standard chow, Lab Diet Rodent Diet 5001 (PMI Nutrition International, St Louis, MO). 

### 2.3. Ventilation-Induced Inflammation and the Isolated Perfused Mouse Lung (IPML) Model

A model of ventilation-induced inflammation was employed as previously described to obtain lung-derived mediators [8, 10]. Using this technique, male 129X1/SVJ mice weighing between 25 and 30 grams were sacrificed and placed on the IPML and mechanically ventilated. Briefly, the pulmonary artery was initially isolated, cannulated, and secured using 4-0 silk. A second cannula was then inserted into the left ventricle and single pass of perfusate (RPMI 1640 lacking phenol red, +2% low endotoxin grade bovine serum albumin; Sigma, St Louis, MO) was utilized to clear the lung of the remaining blood. Subsequently, a continuous reperfusion of the pulmonary circulation was performed using approximately 10 mL of perfusate. This perfusate was used to replace the blood within the pulmonary vascular compartment, while bovine serum albumin was included to maintain the integrity of the pulmonary vessels. Animals were mechanically ventilated with room air for a period of 2 h with a tidal volume (*V*_t_) of 12.5 mL/kg, respiratory rate of 30 breaths/min, positive end expiratory pressure (PEEP) of 3 cm H_2_O while using 5% CO_2_ to maintain the pH of the bicarbonate-buffered RPMI. At the completion of the ventilation protocol, lung perfusate was collected and immediately stored at −80°C. Lung perfusate was pooled and the levels of inflammatory cytokines in lung perfusate were determined using a Millipore Milliplex kit according to the manufacturer's protocol (Millipore, Billerica, MA) for ten inflammation relevant analytes using a multiplex assay. Samples were analyzed using the Luminex xMAP detection system on the Luminex^100^ (Linco Research, St Charles, MO) as per manufacturer's instructions. New non-circulated perfusate media (control perfusate) were used as a blank control in the ELISA as well as a baseline or negative control in subsequent *in vitro* cell culture experiments.

### 2.4. Mouse Liver Endothelial Cell Culture

Mouse liver endothelial cells (MLEC) were a kind gift from Dr. Steven Alexander (Louisiana State Health Sciences Center, Shreveport, LA, USA). MLEC were cultured in (minimal essential media) MEM D-Valine (PromoCell, Heidelberg, Germany) supplemented with 10% fetal bovine serum (FBS), 2 mM L-glutamine (Invitrogen, Burlington, ON), MEM nonessential amino acids (Invitrogen), MEM vitamin mix (Invitrogen), and 1% penicillin/streptomycin (Invitrogen). Cells were passaged twice per week.

### 2.5. Multiplex Enzyme-Linked Immunosorbent Assay (ELISA)

MLECs were seeded in a 24-well plate (6 × 10^4^ cells per well) 2 days prior to the experiment. The confluent MLEC monolayers were challenged for 8 h in a cell culture incubator with 0.25 mL of: (a) control uncirculated perfusate, (b) uncirculated perfusate containing cytomix using equal concentrations of TNF-*α*, IL-1*β* and interferon (IF)-*γ* (10 ng/mL), (which has been used to simulate inflammatory conditions in cell culture [14]), or (c) lung perfusate. These conditioned media were then frozen at −80°C for later analysis. The obtained conditioned media were analyzed with the Millipore Milliplex kit and Luminex xMAP detection system as described above.

### 2.6. Determination of NF-*κ*B Activity in MLEC

MLEC were plated two days prior to experimentation in 6-well (western blot) or 24-well (ELISA) plates at 1.5 × 10^5^ or 6 × 10^4^ cells per well, respectively. Control perfusate or lung perfusate was subsequently applied to MLEC cultures for 30 minutes. Following stimulation, cells were washed three times with cold phosphate buffered saline (PBS) and lysed in a buffer containing 0.5% sodium dodecyl sulfate (SDS), 1 mM ethylenediaminetetraacetic acid (EDTA), 50 mM Tris pH 7.5 plus 1 : 100 Protease Inhibitor cocktail (Sigma, St. Louis, MO). Cell lysates were subsequently boiled and subjected to western blot analysis using an anti-phospho-p65 antibody (Cell Signaling, Beverley, MA, USA) and anti-GAPDH (Cell Signaling, Danvers, MA) as previously described [15]. For the detection of phospho-p65 by ELISA, cells were lysed and processed according to the manufacturers instructions using the Pathscan phospho-p65(Ser536) ELISA kit (Cell Signaling). ELISA results were normalized to the total protein content per well as determined by the micro bicinchoninic acid (BCA) technique (Thermo Scientific, Nepean, ON).

### 2.7. Real-Time Quantitative Polymerase Chain Reaction (qPCR)

1.5 × 10^5^ MLECs were placed on 35 mm dishes 2 days prior to exposure to 0.8 mL of the indicated perfusates (with or without NF-*κ*B inhibitors) for 4 hours at 37°C. Total RNA was extracted from the cells using the RNeasy Plus Mini Kit (Qiagen, Toronto, ON, Canada). 1 *μ*g of total RNA was reverse-transcribed using Superscript III reverse transcriptase (Invitrogen) following the manufacturer's protocol. qPCR was performed as described previously [10] with the exception that the Cq values were determined by linear regression in CFX Manager v2.1 (Biorad, Mississauga, ON). Cq data was exported into qbasePLUS (Biogazelle, Zwijnaarde, Belgium) for quantification of expression and statistical analysis. The gene-specific PCR efficiencies were determined using the “qpcR” package v1.36 in “R” v2.15.0 (http://www.r-project.org/) [16]. The data were fitted to a 5-parameter logistic curve using the smoothing option to determine reaction efficiencies using the Cy0 method. The control perfusate samples were used as the calibrator in each reaction for cultured cells, unventilated control livers were arbitrarily set to 1 for graphing after analysis. The target gene expression was normalized to the *β*-actin, GAPDH, and 18S RNA in all samples. Primer sequences were obtained from RTPrimerDB [17]: *β*-actin ID: 168, IL-6: 3269, TNF-*α*: 3747, CXCL-2: 1068, or CXCL-1 [18], GAPDH: Fwd 5′-CAACGACCCCTTCATTGACCTC-3′ and Rev 5′-CCAATGTGTCCGTCGTGGAT-3′, 18s (a kind gift from Dr. Aaron Cox, Western University, London, ON, Canada): Fwd 5′-ACGATGCCGACTGGCGATGC-3′ and Rev 5′-CCCACTCCTGGTGGTGCCCT-3′.

### 2.8. NF-*κ*B Inhibitors

For experiments involving NF-*κ*B pathway inhibition, cells were preincubated with 15 *μ*M IMD-0354 (Tocris Bioscience, Minneapolis, MN) or 20 *μ*M caffeic acid phenethyl ester (CAPE) (Tocris) for 20 minutes, prior to exposure with lung perfusate that also contained the same concentration of the indicated inhibitor. A short preincubation period was used to ensure the NF-*κ*B pathway would not be activated immediately upon exposure to the inflammatory mediators in the perfusate.

### 2.9. *In Vivo* Model of Ventilation-Induced Inflammation

C57BL/6 mice weighing between 20 and 30 grams were initially anesthetized with ketamine (100 mg/kg) and xylazine (5 mg/kg) and subsequently the left jugular vein and left carotid artery were exposed and cannulated with PE10 tubing which was secured in place with 5-0 silk. The arterial line was used to collect arterial blood samples (60 *µ*L each time) for blood gas measurements (ABL 700, Radiometer, Copenhagen, Denmark), monitor hemodynamics, and deliver fluids (sterile 0.9% NaCl and 100 IU heparin/L) using an infusion pump at a rate of 0.5 mL/100 g/h. The venous line was used to deliver additional ketamine/xylazine anesthetic as needed and to deliver additional fluid (0.5 mL/100 g/h) continuously. Ketamine/xylazine was administered through the venous line to maintain a consistent level of anesthesia and avoid additional unnecessary animal handling. The trachea was exposed and a 14-gauge endotracheal tube was secured with 3-0 surgical silk. Animals were subsequently connected to the Harvard Mini-Vent volume-cycled mechanical ventilator (Harvard Instruments, Saint-Laurent, Canada) with the following parameters: *V*_t_ = 10 mL/kg, PEEP = 3 cm H_2_O, respiratory rate = 150 breaths/min (bpm), and FiO_2_ = 1.0. After 15 minutes of ventilation, animals were assessed for initial inclusion criteria, which consisted of a ratio of arterial partial pressure of oxygen to fractional percentage of inspired oxygen (PaO_2_ : FiO_2_) of >400 mmHg. 

Every fifteen minutes, for the subsequent 240 minutes measurements were taken of peak inspiratory pressure (PIP) blood pressure (BP), heart rate (HR) and recorded while temperature was constantly measured with a rectal probe attached to an Omega Engineering, HH-25TC thermocouple. After 4 h of ventilation, the animals were euthanized with an intravenous overdose of sodium pentobarbital (110 mg/kg). Liver samples were subsequently excised and snap frozen for later RNA extraction using Trizol reagent (Invitrogen) as per the manufacturer's protocol. qPCR was performed on extracted RNA samples as described above.

### 2.10. Statistical Analysis

Groups were analyzed by one-way analysis of variance (ANOVA) (cell culture samples) or Student's *t*-test (livers) using GraphPad Prism v4.03 (GraphPad Software Inc, La Jolla, CA), except qPCR statistics were performed using qbasePLUS's internal statistical analysis by one-way ANOVA (cell culture samples) or Student's *t*-test (livers). Results were considered significant when *P* < 0.05.

## 3. Results 

### 3.1. Generating Lung-Derived Inflammatory Mediators

In order to elicit ventilation-induced lung inflammation in mice and obtain lung-specific mediators in a perfused solution, we ventilated euthanized mice on the IPML apparatus. Analysis of the inflammatory cytokine concentrations in lung-derived perfusate collected at the completion of the mechanical ventilation protocol is shown in Table 1. The concentrations of lung-specific mediators from ventilated mice were comparable to previous observations made by our group using this protocol [10].

### 3.2. Mouse Liver Endothelial Cell Response to Lung-Derived Mediators

**Figure 1:**
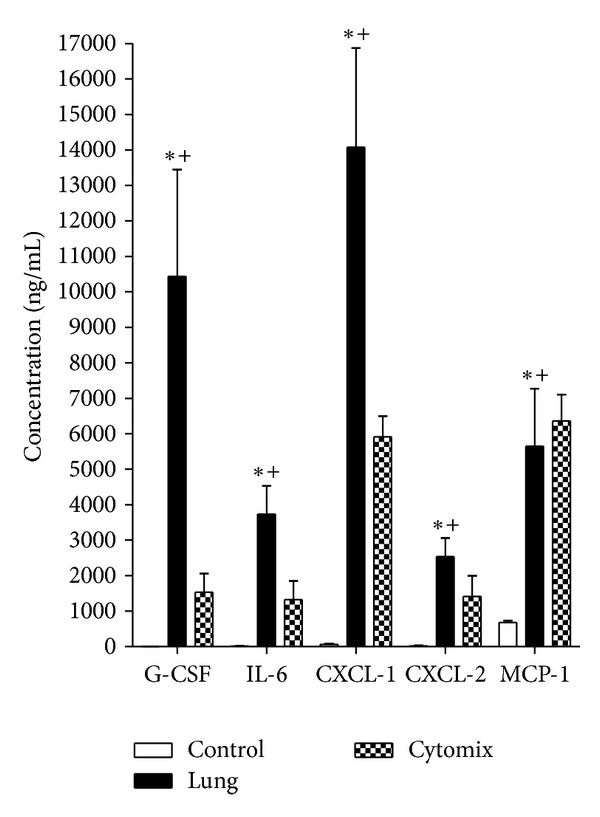
Absolute increase in selected cytokine concentrations in MLEC media. G-CSF, IL-6, CXCL-1, CXCL-2, and MCP-1 concentrations from MLECs exposed to control uncirculated perfusate (open bar), lung perfusate (solid bar), and cytomix (checkered bar). (*P < 0.05 concentration after 8 h incubation on MLEC versus concentration before incubation on MLEC (0 h), ^+^ *P* < 0.05 versus control perfusate after both were incubated on MLEC cultures for 8 h, ±SEM). Where open bars are not apparent (G-CSF, IL-6, CXCL-1, CXCL-2), the increase is too small to print.

MLECs were exposed to control uncirculated perfusate, lung perfusate, or uncirculated perfusate plus cytomix for 8 h. MLECs exposed to lung perfusate expressed significantly greater concentrations of granulocyte colony stimulating factor (G-CSF), IL-6, chemokine (C-X-C motif) ligand 1 (CXCL-1), CXCL-2, and monocyte chemoattractant protein 1 (MCP-1) measured within the conditioned media compared to MLECs exposed to control perfusate and compared to concentrations in the perfusate before incubation on MLECs. The increases in cytokine concentrations is shown in Figure 1. Four of the analytes included in the assay demonstrated no significant change in concentrations after 8 h of incubation with lung perfusate (IF-*γ*, IL-1*β*, IL-10, and TNF-*α*) as compared to the baseline concentrations, while eotaxin decreased significantly from baseline (data not shown). Incubation of MLEC with cytomix (10 ng/mL) demonstrated significant increases in MCP-1, granulocyte macrophage colony stimulating factor (GM-CSF), TNF-*α*, IL-1*β*, and eotaxin, while the remaining analytes demonstrated no significant change from control. 

### 3.3. Lung-Derived Mediator Effects on NF-*κ*B Activation and Gene Expression in MLEC

**Figure 2:**
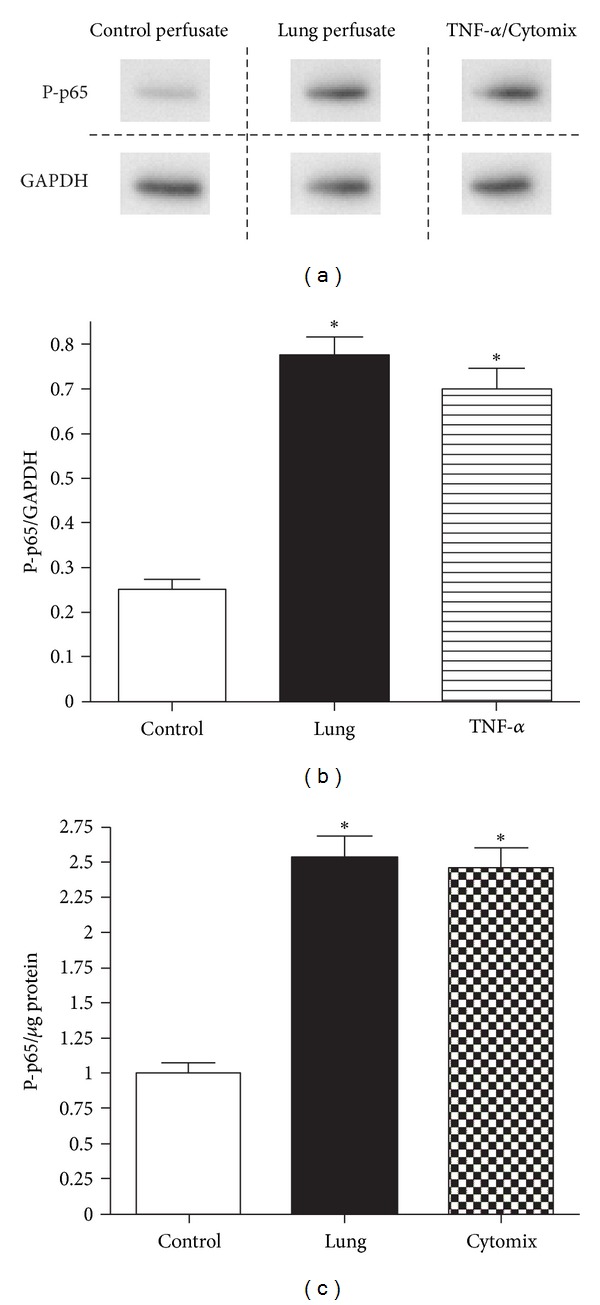
Activation of NF-*κ*B in MLEC exposed to control uncirculated perfusate, lung perfusate, and TNF-*α*/cytomix. (a) Representative western blot and (b) densitometry of P-p65 relative to GAPDH expression. (c) Activation of NF-*κ*B employing phospho-p65 (Ser536) ELISA. (*P < 0.05 versus control, ±SEM).

Based on the observation that incubation with lung perfusate elicited the production of further inflammatory mediators, we investigated the role of the inflammation-relevant NF-**κ**B signaling pathway in this process. Incubation of MLEC with lung perfusate resulted in a significant increase in NF-*κ*B-subunit p65 phosphorylation compared to cells incubated with control perfusate media (Figure 2). Figures 2(a) and 2(b) depict a representative western blot and quantification of phospho-p65 from MLEC stimulated with either lung perfusate or TNF-*α* as a positive control. Similarly, in independent experiments, activation of NF-*κ*B was also confirmed employing an ELISA approach to detect phospho-p65 (Ser536) (Figure 2(c)) with cytomix used as a positive control. p65 phosphorylation, detected by ELISA, was significantly increased in lung perfusate and cytomix exposed cells compared to control perfusate alone. 

**Figure 3:**
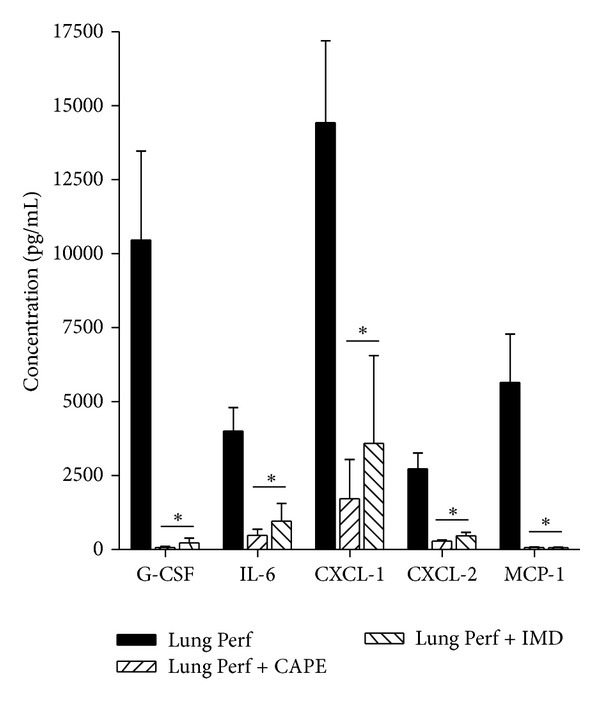
NF-*κ*B inhibitor effect on cytokine concentrations in MLEC-exposed media. G-CSF, IL-6, CXCL-1, CXCL-2, and MCP-1 after 8 h exposure to lung perfusate and treatment with CAPE or IMD. *P < 0.05 versus lung perfusate, ±SEM.

Based on the above observations, two structurally different NF-*κ*B inhibitors, IMD-0354 (IMD) and caffeic acid phenethyl ester (CAPE), were employed. These compounds have previously been determined to interfere with NF-*κ*B activation at two different points along the NF-*κ*B signaling cascade [19, 20]. Initial experiments were performed to determine the effective and minimally cytotoxic concentrations of both inhibitors. The obtained results indicated that IMD and CAPE were effective in suppressing NF-*κ*B activation at 15 and 20 *μ*M, respectively (data not shown). Treating MLEC with either IMD or CAPE significantly mitigated the production of proinflammatory mediators released by MLEC after incubation with lung perfusate as shown in Figure 3. 

**Figure 4:**
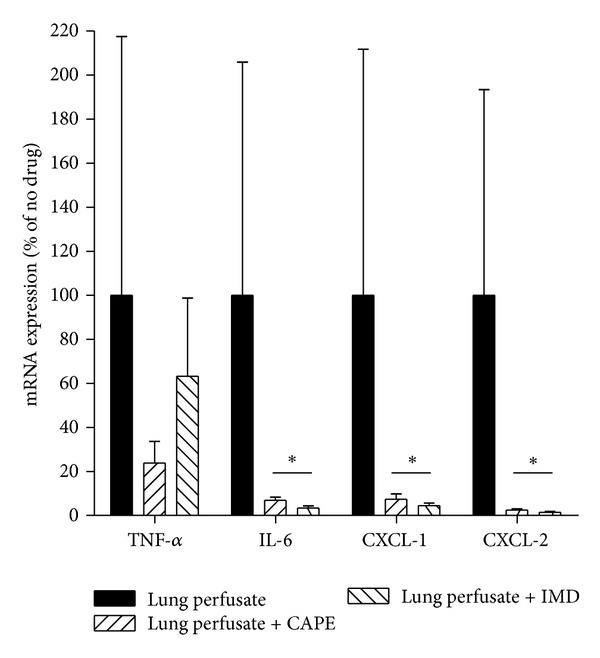
Quantitative PCR of TNF-*α*, IL-6, CXCL-1, and CXCL-2 expressed in MLEC in response to lung perfusate alone or treatment with CAPE or IMD during lung perfusate exposure. *P < 0.05 versus Lung perfusate, ±SEM.

To confirm that these changes occurred at the level of gene transcription, selected mediators were chosen for qPCR analysis in MLECs exposed to lung perfusate (Figure 4). Gene transcription of IL-6, CXCL-1 and CXCL-2 were significantly reduced by treating MLEC with either IMD or CAPE prior to exposure to lung perfusate, whereas neither inhibitor had a significant effect on the gene expression of TNF-*α*, although there was a trend of reduced TNF-*α* expression.

### 3.4. Hepatic Inflammatory Cytokine Expression *In Vivo*

**Figure 5:**
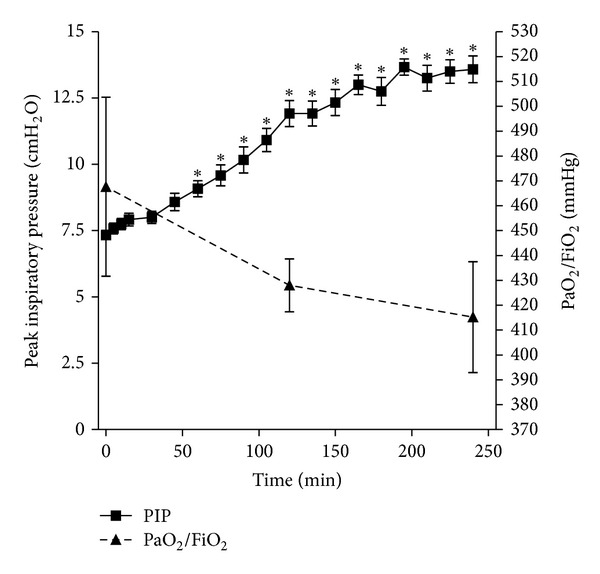
Physiological parameters during *in vivo* ventilation. Peak inspiratory pressure (PIP) (solid line, left axis) was determined every 15 minutes; arterial partial pressure of oxygen over fraction of inspired oxygen (PaO_2_/FiO_2_) (dashed line, right axis) was determined at time 0, 120, and 240 minutes of *in vivo* mechanical ventilation. *P< 0.05 versus time 0, ±SEM.

**Figure 6:**
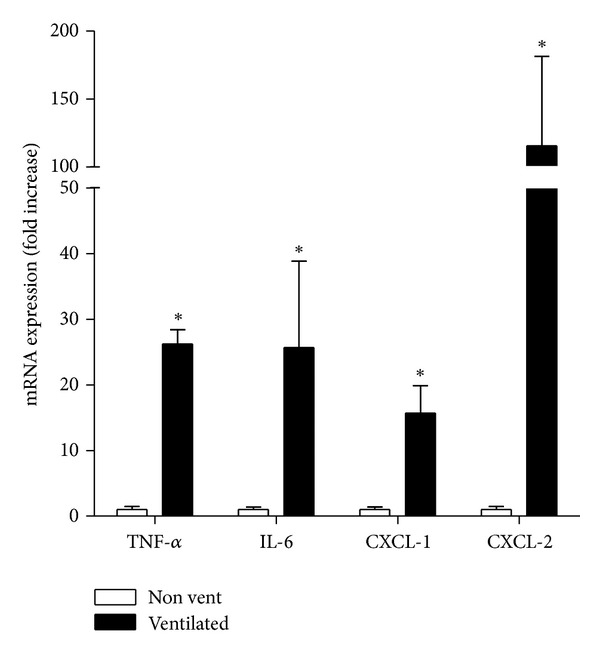
Quantitative PCR for TNF-*α*, IL-6, CXCL-1, and CXCL-2 in liver tissues extracted from nonventilated (nonvent) and *in vivo* mechanically ventilated (ventilated) mice. Values represent fold-increase over expression in nonventilated mice. *P < 0.05 versus non-ventilated mice ±SEM.

Physiological parameters for animals undergoing 2 hours of mechanical ventilation are shown in Figure 5. Over the course of mechanical ventilation, there was a decrease in PaO_2_ at both 120 and 240 minutes of mechanical ventilation compared to the baseline PaO_2_; however, this decrease was not statistically significant. In contrast, the PIP, also shown in Figure 5, increased over the course of ventilation and was significantly increased at 60 minutes and thereafter compared to the baseline (time 0) PIP. Additionally, blood pressure and partial pressure of CO_2_ did not vary significantly from the baseline (data not shown). The lack of a significant change in the majority of these parameters suggested that a significant degree of lung dysfunction was not elicited by this ventilation protocol. Figure 6 depicts the qPCR analysis of selected inflammatory mediators expressed in mouse livers. qPCR demonstrated a significant increase in CXCL-1, CXCL-2, IL-6, and TNF-*α* gene transcription in livers of mechanically ventilated animals compared to non-ventilated controls, a phenomenon consistent with our observations made *in vitro*. 

## 4. Discussion

The results of the current study present a novel finding of an NF-*κ*B-dependent mechanism of proinflammatory cytokine amplification by liver endothelial cells secondary to mechanical ventilation. Previous studies have consistently demonstrated that the NF-*κ*B signaling cascade represents a key regulatory process controlling the transcription of many proinflammatory mediators as it is estimated that over 400 activators of this inflammatory pathway [21] have been identified including physical stress [22], oxidant stress [23], and proinflammatory cytokines [24]. Thus, while it may not be unexpected that lung perfusate obtained from ventilated mice that is rich in multiple proinflammatory cytokines is capable of activating the NF-*κ*B pathway in liver endothelial cells, we highlight unique aspects which we believe are relevant in the context of systemic inflammation subsequent to the initiation of mechanical ventilation. 

**Figure 7:**
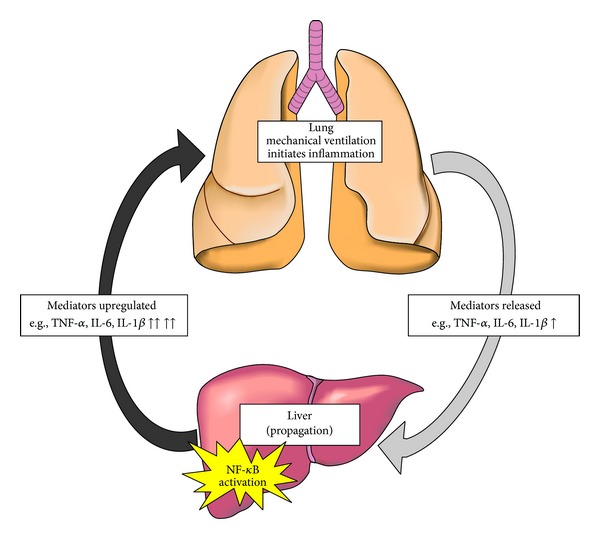
Proposed inflammatory pathway diagram. Inflammation initiated in the lung releases inflammatory mediators (light grey arrow, right side) which then translocate to peripheral organs (e.g., liver). These organs amplify the inflammatory signal, through an NF-*κ*B-dependent pathway, leading to further release of inflammatory mediators, which then travel back to the lung, and/or other peripheral organs (dark grey arrow) where the signal is further propagated in a feed-forward mechanism of acute inflammation.

Firstly, through the use of the IMPL model, we show that *specific* mediators originating from a lung generated in response to mechanical ventilation are capable of inducing NF-*κ*B signaling in endothelial cells of a peripheral organ. The IMPL model allows the pulmonary circulation to be isolated from the systemic circulation, thereby facilitating the collection of mediators generated directly by the lung as a result of mechanical (ventilation) stress. Although other aspects of the IPML model may have contributed to the inflammatory mediators in perfusate, such as surgery and lack of blood, current literature suggests that the vast majority of these mediators are induced by the cell stretch due to ventilation [25–27]. From a clinical standpoint, although the absolute rises in serum cytokines have been directly correlated with outcomes in the setting of ARDS [11], the specific origin of these mediators has been incompletely characterized. Therefore, based on the results of this study we speculate that although the injured lung serves as the primary origin of the systemic inflammatory response, the signal is promptly propagated by peripheral organs in a maladaptive “feed-forward” mechanism of systemic inflammation. Figure 7 depicts an illustration of this pathway. 

Secondly, while this lung-derived perfusate contains elevated levels of multiple inflammatory mediators, equivalent or greater concentrations of cytomix (TNF-*α*, IL-1*β*, IF-*γ*) failed to elicit an equal magnitude of responses. These findings would suggest that the effects observed in our model may be an aggregate effect of multiple mediators present in lung perfusate samples which are generated specifically through the effects of mechanical ventilation. Furthermore, the downstream increase in inflammatory mediators originating from liver cells was not simply a global, nonspecific effect. Rather, although liver endothelial cells are capable of producing a wide spectrum on inflammatory mediators [28], the rise in mediators appeared to be restricted to a significant increase in 5 out of 10 analytes measured including G-CSF, IL-6, CXCL-1, CXCL-2, and MCP-1. Notably, TNF-*α* was not significantly elevated in the cell culture model, although TNF-*α* gene transcription was significantly up-regulated in lung perfusate treated cells. This may be related to the known properties of the TNF-*α* gene which is rapidly transcribed upon stimulation, but has subsequent translation tightly controlled [29]. Although some mediators were not significantly increased upon exposure to the MLEC cultures (IF-*γ*, IL-1*β*, IL-10, and TNF-*α*), this is not to suggest these mediators are not important or do not contribute to inflammation. These findings not only underscore the complexity of the systemic inflammatory response secondary to mechanical ventilation, but also may explain why previous therapeutic interventions targeting isolated cytokines have not resulted in improvement in patient outcomes [30]. 

Using the *in vivo* model of ventilation-induced inflammation highlights several interesting observations. Although the use of mechanical ventilation is obligatory in the setting of ALI and ARDS to maintain host survival, the *in vivo* model adopted in the current study employed the use of mechanical ventilation alone to study its downstream effects on systemic inflammation. Despite the absence of marked changes in host physiology (oxygenation), significant proinflammatory changes were noted in liver tissues suggesting that systemic manifestations of mechanical ventilation may not only occur in the absence of physiological lung dysfunction but that pre-existing lung injury may not be an obligatory requirement for potentially deleterious systemic manifestations. 

Clinical studies in patients with ARDS have consistently demonstrated that stepwise increases in inflammatory cytokines in patients with ARDS have been correlated with greater adverse outcomes [31, 32]. For example, Ranieri et al. showed that patients exposed to protective modalities of MV had lower pulmonary and systemic inflammation compared to patients on conventional ventilation strategies [33]. Furthermore, other studies have also demonstrated that patients ventilated with lower tidal volumes had a lower plasma level of IL-6, as well as soluble TNF-*α* and IL-1 receptor antagonists compared to those ventilated with conventional strategies, thereby providing evidence that mechanical ventilation independently leads to systemic inflammation [11]. The current study adds to the growing body of evidence that injudicious use of mechanical ventilation can contribute adversely toward a maladaptive systemic inflammatory response by peripheral organs, and furthermore, may provide insight into potential mechanism by which therapeutic approaches, such as low tidal volume mechanical ventilation, have been successful in improving patient outcomes.

Our data would suggest that the adoption of either a primary or complementary strategy of mitigating peripheral organ responses early in the course of ARDS through the blockade of maladaptive pathways such as NF-*κ*B signaling in peripheral organs may be an effective approach to consider. Alternately, strategies aimed at minimizing the translocation of lung-derived mediators into the systemic circulation may represent a more “proximal” upstream approach; however, the specific mechanisms responsible for the release of these mediators remains as yet undetermined. 

Although we describe a potential mechanism whereby inflammatory signals originating the in the lung are subsequently amplified by cells of a downstream organ, we recognize that our model does have inherent limitations. Firstly, we chose to utilize liver endothelial cells as the cell type of interest due to the immediate proximity and exposure of this cell layer to lung-derived mediators which may circulate *in vivo*. Therefore, our findings are limited to this specific cell type and we have not accounted for the contribution of other tissue specific cells within the liver such as hepatocytes or Kuppfer cells, for example. The contribution of other cell types from liver and other organs may account for why we did not observe significant increases in several mediators previously shown to be important in patient outcomes (e.g., IL-1*β*, TNF-*α*). Nonetheless, the use of whole liver tissues employed in the *in vivo* model of mechanical ventilation indicates that increases in IL-6, for example, may be expressed throughout the liver and not restricted to any one cell type. Secondly, our investigation focused primarily on proinflammatory effect, the contribution of anti-inflammatory mediators in this process may also be important to evaluate in future studies. Thirdly, it remains unknown whether similar links exist between the lung and other downstream organs such as the kidneys, heart or brain and whether an amplification of inflammatory mediators from theses other systemic organs contribute to a greater or lesser extent toward systemic inflammation. Whether the NF-*κ*B signaling cascade represents a common pathway of proinflammatory signaling within each organ or whether other organ specific proinflammatory signaling pathways exists remains to be characterized. Future studies to determine the generalizability of our findings beyond a single downstream organ are therefore warranted.

In the current study, we demonstrate that inflammatory mediators generated by the lung in response to mechanical ventilation decompartmentalize to the systemic circulation in a murine model of ventilator-induced inflammation. Subsequently, we show that the levels of these inflammatory mediators are significantly amplified upon exposure to liver endothelial cells thereby resulting in a maladaptive upregulation of the systemic inflammatory response. The results of *in vitro* experiments illustrating this phenomenon are further confirmed in an *in vivo* model of ventilation induced inflammation whereby a significant increase in transcriptional activity in these mediators is observed in the liver. Ultimately, we show that the propagation of the systemic inflammatory response by the liver occurs through an NF-*κ*B-dependent mechanism and that inhibition of this signaling pathway can, in part, mitigate these responses. The significance of these findings will require further studies to determine whether blockade of the NF-*κ*B pathway in peripheral organ tissues would provide a rational means of therapeutic intervention.

## Figures and Tables

**Table 1:** Cytokine concentration in lung perfusate samples collected from animals sustaining ventilator-induced inflammation at the completion of 2 hours of mechanical ventilation using the isolated perfused mouse lung model. New uncirculated perfusate was used as the blank control for the ELISA. Values represent mean ± SEM.

Mediator	Increase over control (pg/mL)
G-CSF	21.87 ± 4.57
IL-6	267.81 ± 28.35
KC	356.38 ± 57.98
MIP2	198.70 ± 12.24
MCP-1	2.01 ± 1.54
TNF-*α*	33.16 ± 4.55
IFN-*γ*	1.44 ± 0.88
IL-1*β*	0.51 ± 0.35
IL10	0.95 ± 0.62
Eotaxin	17.46 ± 2.93
